# Editorial: Editor’s feature: negative findings in pharmacogenetics and pharmacogenomics volume II

**DOI:** 10.3389/fphar.2025.1658876

**Published:** 2025-07-15

**Authors:** José A. G. Agúndez, Elena García-Martín, Luis Abel Quiñones

**Affiliations:** ^1^ Universidad de Extremadura, Institute of Molecular Pathology Biomarkers, University of Extremadura, Cáceres, Spain; ^2^ Laboratory of Chemical Carcinogenesis and Pharmacogenetics, Department of Basic-Clinical Oncology (DOBC), Faculty of Medicine and Department of Pharmaceutical Sciences and Technology, Faculty of Chemical and Pharmaceutical Sciences, University of Chile, Santiago, Chile; ^3^ Latin American Network for Implementation and Validation of Clinical Pharmacogenomic Guidelines (RELIVAFCYTED), Santiago, Chile

**Keywords:** pharmacogenomics (PGx), pharmacokinetics and drug metabolism (PDM), adverse drug events, genotyping, negative findings

This second volume of the Research Topic “*Negative Findings in Pharmacogenetics and Pharmacogenomics*” brings together seven original research articles contributed by a total of 50 authors from diverse institutions and countries. As in the first edition (Agúndez et al.), the aim is to provide a platform for well-designed studies that, despite yielding negative or non-significant results, offer valuable insights for the scientific community and contribute to a more balanced and transparent evidence base. The publication of negative findings remains essential to counteract publication bias, which can distort the scientific record, inflate expectations, and lead to unnecessary duplication of research efforts. In the context of pharmacogenetics and pharmacogenomics, where expectations for clinical translation are high and study replication is often challenging due to population heterogeneity and cost, the transparent reporting of negative results is particularly valuable. Negative results, when rigorously obtained and clearly reported, help refine hypotheses, rule out confounding variables, and improve the precision of predictive models for drug response and toxicity. They also prevent overestimation of genetic effect sizes and reduce the likelihood of pursuing clinically irrelevant biomarkers. Unfortunately, many studies with negative results, even those published in high-impact medical journals often lack critical information such as *a priori* power calculations, confidence intervals, or clearly defined primary outcomes. This omission limits the ability of readers to assess the validity and clinical relevance of the findings. In this context, the studies included in this volume were selected for their methodological and their commitment to transparent reporting.

Tsironi et al. assessed the influence of four genetic variants (*CYP3A4* rs2242480, and rs4986910; *ABCB1* rs1128503, and rs2229109) on tacrolimus dose-adjusted trough concentrations in kidney transplant recipients over a 1-year period. The authors found no significant associations between these variants and tacrolimus pharmacokinetics or dose requirements. Advanced statistical and machine learning analyses confirmed the lack of predictive value of these genotypes for tacrolimus exposure. These findings align with previous studies reporting inconsistent or null associations for these variants, particularly in European populations. The study underscores the complexity of tacrolimus pharmacogenetics and the need for broader genomic approaches and larger, multi-ethnic cohorts to identify clinically actionable markers.

Lindarte et al. evaluated the impact of *CYP3A5* polymorphisms on tacrolimus pharmacokinetics in liver transplant recipients. The authors observed that CYP3A5 expressers (carriers of the *1 allele) required higher tacrolimus doses and exhibited lower dose-adjusted trough concentrations (C_0_/dose) compared to non-expressers. These differences persisted over a 2-year follow-up period. The study provides valuable longitudinal data in a Latin American population, a group underrepresented in pharmacogenetic research. The findings reinforce the relevance of *CYP3A5* genotyping in optimizing tacrolimus dosing. This study is relevant to discussions of negative or non-confirmatory findings because, despite confirming expected pharmacokinetic differences by CYP3A5 genotype, it did not demonstrate a clear impact on clinical outcomes such as graft survival or rejection rates. Its inclusion underscores the gap between genetic associations and their clinical translation, especially in underrepresented populations.

Leibold et al. investigated the association between *CYP3A5* variants (*3, *6, and *7) and blood pressure traits, including systolic and diastolic blood pressure, mean arterial pressure, and hypertension diagnosis. No major associations were found in the overall population. Although minor statistically significant associations were observed in white participants (e.g., CYP3A5*3 with slightly higher DBP), the effect sizes were, and no associations were replicated in other ancestral groups. These results suggest that *CYP3A5* polymorphisms are unlikely to have clinically relevant effects on blood pressure regulation in the general population, highlighting the importance of distinguishing statistical from clinical significance in large-scale genomic studies.

The study by Gándara-Mireles et al. investigated the influence of individual characteristics and three single-nucleotide variants (SNVs) in the *GRIA1* and *NFATC2* genes on adverse events associated with L-asparaginase (L-Asp) in children with acute lymphoblastic leukemia. The authors found a significant association between the *GRIA1* rs4958351 AA genotype and both the occurrence of adverse events and the development of anti-L-Asp antibodies. No associations were observed for *GRIA1* rs6889909 or *NFATC2* rs6021191. The lack of association for these variants, despite prior interest, highlights the complexity of pharmacogenomic prediction and the importance of reporting null results Additionally, low body mass index and T-cell immunophenotype were identified as clinical risk factors for pancreatitis and hypersensitivity reactions. These findings highlight the relevance of pharmacogenetic and clinical profiling in predicting L-Asp toxicity.

Escalante et al. explored whether miR-92a-3p modulates the expression of key genes involved in resistance to FOLFOX chemotherapy in colorectal cancer. Using SW480 and SW620 cell lines, the authors assessed the impact of miR-92a-3p on the expression of DNA repair genes (*ERCC1, ERCC2,* and *XRCC1*) and genes coding for enzymes in the fluoropyrimidine pathway (*DPYD, TYMS,* and *MTHFR*). While miR-92a-3p altered mRNA levels of ERCC2 and XRCC1 in SW620 cells, no changes were observed at the protein level. The study also found no evidence of β-catenin activation or epithelial-mesenchymal transition (EMT) induction These findings indicate that miR-92a-3p is unlikely to mediate FOLFOX resistance through epigenetic regulation of the evaluated biomarkers, suggesting that its role in chemoresistance may involve alternative molecular targets or regulatory pathways. This underscores the need to further explore the epigenetic landscape contributing to FOLFOX response, including other microRNAs, DNA methylation and chromatin-modifying mechanisms.

The study by Hong et al. explored whether key hepatic nuclear receptors—FXR, PXR, LXR, and PPARα—regulate the expression of human arylamine N-acetyltransferase 2 (NAT2), an enzyme known for its role in xenobiotic metabolism. Using cryopreserved human hepatocytes, the authors found that activation of FXR, PXR, and LXR did not alter NAT2 mRNA levels. PPARα activation led to a statistically significant, although biologically marginal decrease in NAT2 expression. These findings suggest that the nuclear receptors tested are not major transcriptional regulators of NAT2 in hepatocytes.

González-Iglesias et al. analyzed the impact of *UGT1A1* polymorphisms on liver biochemical parameters in 773 healthy volunteers participating in bioequivalence trials. Individuals with intermediate or poor metabolizer phenotypes (based on UGT1A1*80+*28 and *6 alleles) showed elevated bilirubin levels, consistent with Gilbert’s syndrome (GS), but no alterations in liver enzyme levels or other biochemical markers. The findings support the inclusion of individuals with UGT1A1-related hyperbilirubinemia in clinical trials, as their liver function remains within normal limits. This work challenges the routine exclusion of individuals with GS from early-phase studies.

In sum, this second volume reaffirms that negative and non-confirmatory findings, when derived from well-powered and methodologically rigorous studies, are essential contributions to scientific progress. Such results clarify the boundaries of pharmacogenetic associations, prevent overinterpretation of preliminary signals, and guide future research toward more fruitful directions. They also underscore the complexity of gene–drug interactions and the need for broader, multi-omic approaches. Importantly, they highlight the value of including diverse populations and real-world variability in research. We hope that this Research Topic encourages researchers, reviewers, and editors to recognize negative results as integral to the advancement of pharmacogenetics and pharmacogenomics. Only by embracing the full spectrum of evidence—positive, negative, and inconclusive—can we build, equitable, and clinically meaningful foundation for personalized medicine.

